# Sleep disorder, an independent risk associated with arterial stiffness in menopause

**DOI:** 10.1038/s41598-017-01489-7

**Published:** 2017-05-15

**Authors:** Yang Zhou, Ruwei Yang, Changbin Li, Minfang Tao

**Affiliations:** Department of Gynecology and Obstetrics, Shanghai Jiao Tong University Affiliated Sixth People’s Hospital, 600 Yishan Road, Shanghai, 200233 People’s Republic of China

## Abstract

As women age and go through menopause, they suffer a higher incidence of sleep disorder, cardiovascular morbidity and mortality. In addition, evidences suggested that sleep disorder was an important pathological indicator for coronary heart disease. However, the relationship between different menopausal status, sleep disorder and cardiovascular diseases was unclear. Thus, we aim to assess the association between sleep disorder with arterial stiffness in females of 40–60 years free of cardiovascular diseases through self-administered Pittsburgh Sleep Quality Index (PSQI) and brachial-ankle pulse wave velocity (baPWV). Logistic regression revealed that sleep disorder (PSQI score ≥ 8) was an independent indicator for higher risk of elevated arterial stiffness (baPWV ≥ 1465.5 cm/s, upper tertile) beyond other established cardiovascular confounders in peri-postmenopause (OR 2.83, 95% confidence interval (CI) 2.00–4.00, p < 0.001), but not in premenopause (OR 1.67, 95% CI 0.71–3.90, p = 0.223). Collectively, it clearly indicates that sleep disorder in menopausal women is of prominent value to predict arterial stiffness.

## Introduction

Menopause is a critical physiological stage of women’s life with various complaints and distresses due to the decline of ovarian hormones^1^. Besides vasomotor symptoms (VMS), such as hot flushes/night sweats, sleep disorder is also one of the most common menopausal symptoms affecting about 50% to 80% of middle-aged women^2^. Additionally, menopause makes women more subjected to higher incidence of cardiovascular morbidity and mortality in the long run^3^.

Both sleep and menopause account for one-third of female’s whole life span, which are of great concern for women’s life quality and long-term health. Women who experience poor sleep are more vulnerable to diseases. Previous evidences have supported that sleep disorder was indicative of higher risks in cardiovascular diseases^4^. Various studies have investigated the association of sleep duration and sleep quality with arterial stiffness^5, 6^. However, the subjective sleep duration variablely associated with arterial stiffness, differing from studies^5, 6^, and the evidence of the relationship between sleep duraion and cardiovascular risks for women was weaker and less conclusive than men^7^. Purely single self-reported sleep quality and duration may result in subjective bias, while the PSQI scale is a realtively more reliable, validated instrument for evaluating sleep quality^8^.

Pulse wave velocity has been purported as a biomarker directly related to vessel stiffness that has the potential to provide siginals on early vascular aging and predict cardiovascular events. It is most often determined through pulse wave velocity (PWV) between two arterial sites^9^. Compared with carotid-femoral pulse wave velocity, brachial-ankle pulse wave velocity (baPWV) is more applicable to clinical routine as a measurement for peripheral arterial stiffness worldwide due to its easy methodology, good reproducibility and non-invasive features^10^.

As the burden of cardiovascular diseases (CVD) in women after menopause is increasing^3^, highlighting the need for middle-aged women-based research designed to disentangling the complex interactions among sleep disorder, menopause and cardiovascular health.

However, little evidence has been avalible to explore the relationship between sleep disorder and arterial stiffness in different stages of menopause in Chinese women through PSQI and baPWV measurement. In this study, we aim to investigate this association in middle-aged Chinese women, and to identify the potential predicting value of sleep disorder for arterial stiffness in different menopausal status.

## Results

### Participants

Among the 1904 participating subjects, 1647 (86.5%) of these were eligible. The basic characteristics between two-score based groups (PSQI ≥ 8, PSQI < 8) were presented in Table 1. Statistical significance of these variables existed between two groups (p < 0.05). Participants were on average 50.29 ± 5.95 years of age. The mean baPWV value was 1341.5 ± 231.7 cm/s. Subjects with sleep disorder tended to be older, obese, menopausal, and had higher blood pressure, waist-hip ratio. Additionally, lipid and glucose disorder, less employed, less income and more parity were also observed in group of sleep disorder (PSQI ≥ 8) (all p < 0.05). In our cross-sectional study, 38.2% reported sleep disturbance. There was ascending trend in the prevalence of sleep disorder among different menopausal groups, with 15.7% in premenopause increasing to 30.3% in late postmenopause (p < 0.001).

**Table 1:** Comparison of variables associated with two–score based groups.

Variables	PSQI < 8	PSQI ≥ 8	total	p
	n = 1017	n = 630	n = 1647	
Age (years)	49.3 ± 5.92	51.8 ± 5.70	50.29 ± 5.95	<0.001
BMI (Kg/m^2^)	23.01 ± 2.94	23.43 ± 3.27	23.18 ± 3.17	0.006
Waist–hip ratio (ratio)	0.82 ± 0.07	0.84 ± 0.07	0.83 ± 0.07	<0.001
SBP (mmHg)	118 ± 15	128 ± 18	121 ± 16	<0.001
DBP (mmHg)	72 ± 9	77 ± 10	74 ± 10	<0.001
Pulse pressure (mmHg)	46 ± 9	50 ± 11	48 ± 10	<0.001
Heart rate (beats/min)	70.10 ± 10.37	72.16 ± 11.30	71.77 ± 37.99	<0.001
Triglycerides (mmol/L)	1.35 ± 1.15	1.53 ± 1.14	1.47 ± 1.23	0.019
Total cholesterol (mmol/L)	5.25 ± 1.05	5.40 ± 1.15	5.27 ± 1.17	<0.001
HDL (mmol/L)	1.53 ± 0.40	1.49 ± 0.36	1.52 ± 0.40	0.015
LDL (mmol/L)	2.98 ± 0.78	3.18 ± 0.86	3.05 ± 0.82	<0.001
FBG (mmol/l)	5.31 ± 1.14	5.60 ± 1.30	5.41 ± 1.20	<0.001
BaPWV (cm/s)	1274.9 ± 194.4	1447.2 ± 246.0	1341.5 ± 231.7	<0.001
Menopausal status				<0.001
Premenopause, n (%)	416 (80.8%)	99 (19.2%)	515 (31.3%)	<0.001
Perimenopause, n (%)	215 (56.9%)	163 (43.1%)	378 (22.9%)	0.031
Early postmenopause, n (%)	202 (53.3%)	177 (46.7%)	379 (23.0%)	<0.001
Late postmenopause, n (%)	184 (49.1%)	191 (50.9%)	375 (22.8%)	<0.001
Marital status				0.627
Married, n (%)	1008 (80.0%)	626 (20.0%)	1634 (92.1%)	0.704
Single/Separated, n (%)	9 (61.7%)	4 (38.3%)	13 (7.9%)	0.787
Divorced/Widowed				
Education, n (%)				0.100
Illiteracy	93 (57.8%)	68 (42.2%)	161 (9.8%)	0.273
Primary	79 (54.9%)	65 (45.1%)	144 (8.7%)	0.057
Junior high	280 (61.1%)	178 (38.9%)	458 (27.8%)	0.570
Senior high	269 (60.7%)	174 (39.3%)	443 (26.9%)	0.450
College	275 (68.6%)	126 (31.4%)	401 (24.3%)	0.002
Postgraduate or above	21 (52.5%)	19 (47.5%)	40 (2.4%)	0.201
Employment status, n(%)				<0.001
Work	676 (66.7%)	338 (33.3%)	1014 (61.6%)	<0.001
Departure	139 (61.8%)	86 (38.2%)	225 (13.7%)	1.000
Retirement	202 (49.5%)	206 (50.5%)	408 (24.8%)	<0.001
Income (RMB/month), n (%)				<0.001
<1000	223 (57.3%)	166 (42.7%)	389 (23.6%)	0.046
1000–3000	190 (54.8%)	157 (45.2%)	347 (21.1%)	0.003
3000–5000	258 (64.7%)	141 (35.3%)	399 (24.2%)	0.169
5000–10000	177 (63.9%)	100 (36.1%)	277 (16.8%)	0.419
>10000	169 (71.9%)	66 (28.1%)	235 (14.3%)	0.001
Parity, n (%)				<0.001
0	29 (80.6%)	7 (19.4%)	36 (2.2%)	0.109
1–2	923 (63.0%)	542 (37.0%)	1465 (88.9%)	0.004
≥3	65 (44.5%)	81 (55.5%)	146 (8.9%)	<0.001
History of disease				
Hypertension, n (%)	165 (43.2%)	217 (56.8%)	382 (23.2%)	<0.001
Diabetes mellitus, n (%)	93 (42.5%)	126 (57.5%)	219 (13.3%)	<0.001

Data were expressed as mean ± SD or frequencies. All data were normally distributed. Age, height, BMI, WHR, SBP, DBP, PP, HR, TC didn’t satisfy the homogeneity of variance, and were analyzed by Mann–Whitney U test. Other continuous variables were determined by independent–Samples T tests. Ordered categorical variables were computed by Wilcoxon rank sum test, while unordered two–categorical variables were tested by χ^2^ tests.

### Age, menopause and baPWV

**Figure 1:**
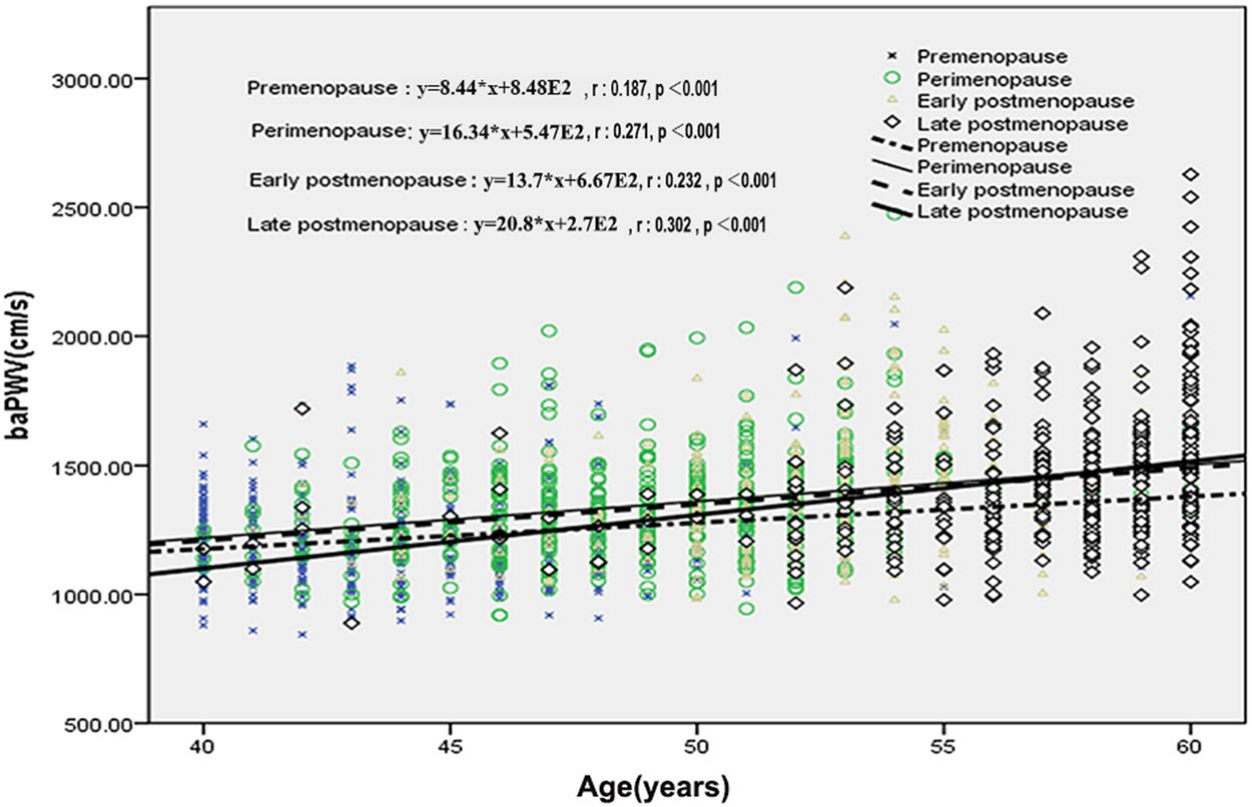
Correlation between age and baPWV in different menopausal status.

As shown in Fig. 1, we found that the slope of the regression line for baPWV plotted against age was steeper in late-postmenopausal, perimenopausal women, early postmenopausal women than in premenopausal women. There was a significant relationship between baPWV and age in each subgroups, including premenopausal (r = 0.187, p < 0.001), perimenopausal (r = 0.271, p < 0.001), early postmenopausal (r = 0.232, p < 0.001), late postmenopausal (r = 0.302, p < 0.001) women. As to the strength of the correlation between age and baPWV, late post menopause > perimenopause > early postmenopause > premenopause. It suggests that menopause per se aggravates the age-related increase in arterial stiffness.

### Prevalence of Sleep disorder, menopause and baPWV

**Figure 2:**
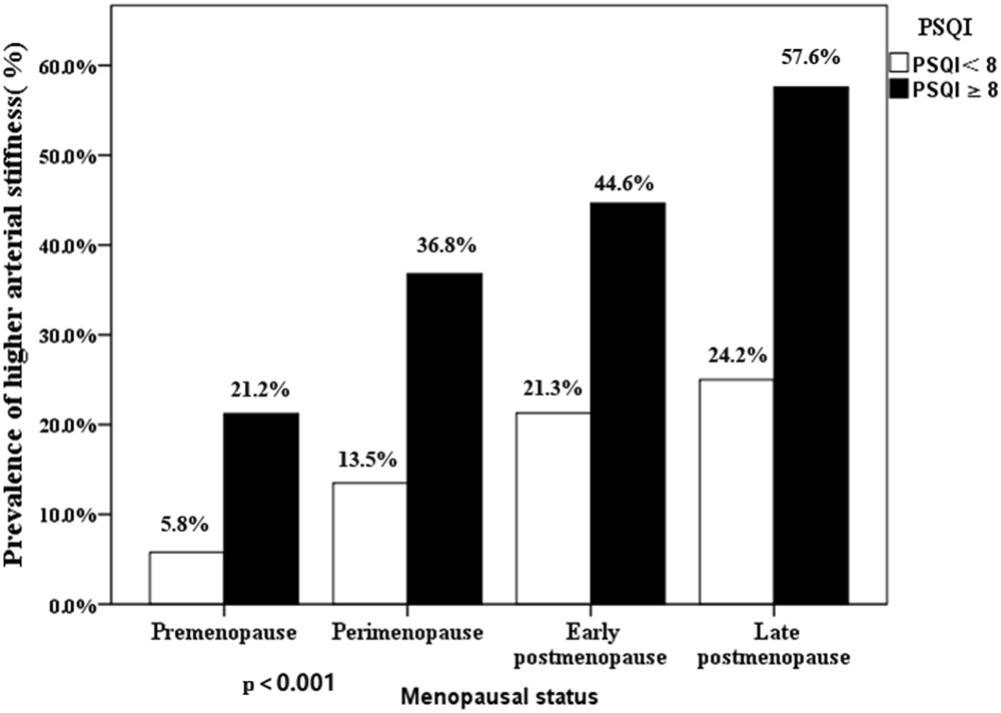
Prevalence of higher arterial stiffness in participants with different combinations of two–score based PSQI and menopausal status.

Regarding to the prevalence of arterial stiffness, in late postmenopause, subjects with sleep disorder had the highest prevalence (57.6%) of arterial stiffness. Participants with sleep disorder all had higher prevalence of increased arterial stiffness than without sleep disorder in the same menopausal status group (p < 0.001) (data not shown). On the whole, with progress of menopause, we found increase in the prevalence of elevated arterial stiffness either in sleep disorder group or without sleep disorder group (Fig. 2).

### Independent risk factors for sleep disorder and baPWV

As shown in Table 2, independent determinants of sleep disorder were age (OR 0.96, 95% CI 0.93–0.99), baPWV (OR 1.00, 95% CI 1.00–1.00), parity and menopause.

**Table 2:** Independent factors for sleep disorder by logistic regression.

Factors	OR (95% CI)	P
Age (years)	0.96 (0.93–0.99)	0.011
BaPWV (cm/s)	1.00 (1.00–1.00)	<0.001
Diabetes Mellitus (yes)	1.66 (1.14–2.48)	0.013
Parity		
0	Ref.	0.001
1–2	2.71 (1.08–6.73)	0.032
≥3	5.17 (1.92–13.95)	0.001
Menopause status		
Premenopause	Ref.	<0.001
Perimenopause	2.82 (1.99–3.99)	<0.001
Early postmenopause	2.93 (1.94–4.43)	<0.001
Late postmenopause	3.16 (1.1–5.21)	<0.001

Covariates: BMI, WHR, SBP, DBP, PP, hypertension, diabetes mellitus, TG, TC, HDL, LDL, FBG, menopause, income, employment status, parity. OR, Odds ratio; CI, confidential interval. Only displayed p < 0.05

For the total subjects, logistic regression identified the independent risk factors for higher arterial stiffness as PSQI ≥ 8 (OR 2.54, 95% CI 1.85–3.47), age (OR 1.16, 95% CI 1.12–1.20), TG (OR 1.22, 95% CI 1.06–1.41), systolic blood pressure(SBP) (OR 1.10, 95% CI 1.09–1.12), fasting blood glucose (FBG) (OR 1.15, 95% CI 1.01–1.06), body mass index (BMI) (OR 0.91, 95% CI 0.87–0.97), heart rate (OR 1.05 95% CI 1.03–1.06), associations persisted adjusting for CVD risk factors mentioned above (Table 3).

**Table 3:** Multivariate adjusted OR and 95% CI for higher arterial stiffness.

Factors	Unadjusted OR (95% CI)	p	Adjusted OR (95% CI)^a^	p
Age (years)	1.16 (1.12–1.20)	<0.001	1.16 (1.12–1.20)	<0.001
SBP (mmHg)	1.10 (1.08–1.12)	<0.001	1.10 (1.09–1.12)	<0.001
Heart rate (bpm)	1.05 (1.03–1.06)	<0.001	1.05 (1.03–1.06)	<0.001
FBG (mmol/L)	1.15 (1.01–1.31)	0.035	1.15 (1.01–1.31)	0.044
BMI (Kg/m^2^)	0.91 (0.89–0.97)	0.001	0.91 (0.87–0.97)	0.004
PSQI ≥ 8 (yes)	2.54 (1.85–3.47)	<0.001	2.54 (1.85–3.47)	<0.001
TG (mmol/L)	1.22 (1.06–1.41)	0.007	1.22 (1.06–1.41)	0.040

^a^Adjusting for BMI, WHR, SBP, DBP, PP, hypertension, diabetes mellitus, TG, TC, HDL, LDL, FBG, menopause, income, employment status, parity, marital status.

We further investigate the role of menopause in sleep disorder-arterial stiffness relation in multi-covariates adjusted model (Table 4). Next, the total subjects were divided into two groups: premenopause and peri-postmenopause. However, in premenopause, PSQI ≥ 8 (OR 1.67, 95% CI 0.71–3.90 p = 0.223) was no longer the independent indicator for arterial stiffness, while showed significant difference in peri-postmenopause (OR 2.83, 95% CI 2.00–4.00, p < 0.001). In addition, there was no association of BMI and TG, hypertension with arterial stiffness in premenopause (Table 4). Taken together, our study put forward that in peri-postmenopausal women, sleep disorder serves as a vital determinant for higher arterial stiffness.

**Table 4:** Independent risk factors for arterial stiffness by menopause in a multi –covariates adjusted model.

Menopausal status	Variables	OR (95% CI)	P
Premenopause	Age (years)	1.17 (1.08–1.28)	<0.001
	Heart rate (beats/min)	1.06 (1.02–1.09)	0.003
	SBP (mmHg)	1.09 (1.06–1.24)	<0.001
	Hypertension n, (%)	1.58 (0.62–4.02)	0.327
	PSQI ≥ 8 (yes)	1.67 (0.71–3.90)	0.223
	BMI, Kg/m^2^	0.90 (0.80–1.02)	0.113
	TG (mmol/L)	1.21 (0.88–1.66)	0.237
(Peri–)Menopause	Age (years)	1.15 (1.10–1.20)	<0.001
	Heart rate (beats/min)	1.05 (1.03–1.07)	<0.001
	SBP (mmHg)	1.11 (1.09–1.13)	<0.001
	Hypertension, n (%)	1.50 (1.02–2.21)	0.042
	PSQI ≥ 8 (yes)	2.83 (2.00–4.00)	<0.001
	BMI (Kg/m^2^)	0.93 (0.87–0.98)	0.012
	TG (mmol/L)	1.25 (1.07–1.47)	0.006

Covariates: BMI, WHR, SBP, DBP, PP, hypertension, diabetes mellitus, TG, TC, HDL, LDL, FBG, menopause, income, employment status, parity. OR, Odds ratio; CI, confidential interval. Only displayed p < 0.05.

## Discussion

Previous studies have supported that sleep disorder was associated with increased arterial stiffness in patients with type 2 diabetes mellitus^11^, ischemic stroke^12^ and obstructive sleep apnea (OSA)^13^. In addition, various studies have found that subjective extreme sleep duration and sleep quality affected vascular aging in the general population, especially in male^4, 5, 14^.

Furthermore, considerable evidence has suggested the association between sleep disorder with menopause^3, 15^, arterial stiffness with menopause^16^, sleep disorder with arterial stiffness^11–13^. However, few corresponding researches were focusing on the interaction with menopause, sleep disorder and arterial stiffness. To the best of our knowledge, there has only been one small-sample cross-sectional study on the relationship between sleep disturbance and arterial stiffness in menopausal women^13^. However, the study subjects included only menopausal women lacking of premenopausal control subjects, and confined to OSA without considering other sleep abnormalities. In addition, OSA, a certain type of intrinsic sleep disorder, has already been proven associated with arterial stiffness^17^. In contrast, our study recruited subjects aged 40–60 covering premenopause, perimenopause and postmenopause so that we can better compare the impact of menopause on the independent risk of sleep disorder associated with arterial stiffness. Moreover, we evaluated the sleep quality by PSQI, which involves various reasons causing sleep abnormalities, especially for menopause-related sleep, a so-called “domino effect” generated by VMS, depressed mood, psychological factors and etc.^18^.

After adjusting for established cardiovascular and demographic risk factors, we interestingly found that sleep disorder (PSQI ≥ 8) was associated with arterial stiffness in peri-postmenopausal women. Accordingly, we suggest that sleep disorder may have some direct influence on arterial stiffness independent of cardiovascular risk factors. The potential mechanisms linking sleep disorder and arterial stiffness possibly can be boiled down into endocrine or metabolic disruption^19^, sympathetic activation^20^, and inflammation and coagulation pathways^21, 22^.

Decline of transmitters such as melatonin, 5-hydroxytryptaminethermoregulatory changes, deregulation in circadian rhythms and sleep-wake cycle, oxidative stress and inflammatory response, caused by the drop of estrogen in menopause, are correlated with the occurrence of poor sleep^23–25^. Additionally, the lower estrogen is permissive to hot flash^26^ and depression^18^, which have been linked with both sleep disorder for women. Activation of pro-inflammatory pathway leading to endothelial function impairment dually mediated by sleep disorder and menopause may present a mechanism that sleep disorder plays an independent detrimental role in arterial stiffness in menopause.

On one hand, withdraw of the role in protecting cardiovascular system by estrogen after menopause may directly cause endothelial function impairment, on the other hand, menopause through triggering sleep disorder indirectly mediates cardiovascular health. Overall, menopause may serve as an incremental role of sleep disorder for higher baPWV. Thus we indicate that the increasing prevalence of sleep disorder is a key contributor to the burgegoing epidemic of arterial stiffness in women, while peri-postmenopause marks this increase in sleep disorder.

Other classical independent factors such as older age, BMI, fasting blood glucose, higher TG for arterial stiffness were compatible with previous study^5, 27–30^.

Although diabetes serves as strong established determinant for CVD^30^ and closely associated with sleep disorder in menopausal women, we did not find that the diabetes effect was as significant as sleep disorder, while fasting blood glucose show the significant independent relationship with elevated arterial stiffness. The result was consistent with one previous study^5^. This may be related to a lower prevalence of diabetes among women (13.3%). In addition, some studies have reported that increased arterial stiffness was not only associated with subjects in diabetes mellitus but also in healthy subjects with impaired fasting glucose^30^ or high-normal glucose level^31^. This may partially explain why it’s FBG not diabetes mellitus was independently associated with arterial stiffness.

In our study, there was an ascending trend in prevalence of higher arterial stiffness with menopause and the advancing duration of menopause. As we all know, aging is a prominent index for arterial stiffness, while we confirmed that menopause augmented the age-related arterial stiffness, which supported the view that menopause per se may elevate the arterial stiffness measuring by baPWV^32–34^ due to impact of the depletion of the ovarian hormones after menopause on vascular aging by breakage and denaturation of elastin production and increasing collagen deposition in arteries^35^. Thus, it may possibly﻿ be proposed to be used as the effective instrument to herald the cardiovascular and all-cause mortality in peri-post menopausal women.

In this cross-sectional study, 38.2% reported sleep disorder (PSQI ≥ 8), and it was higher than our previous study, which reported 33.2% in 2014^15^. Sample selection may attribute to the discrepancy, however,﻿ our finding was﻿ consistent with one from the United States reporting 38% sleep disorder with 40–55 years old women, which was a multiethnic, community-based sample^36^. From what we can imply that more stress stemming from modern society and family in China in recent years resulted in more sleep problem in females, catching up with the prevalence of the developed countries. The prevalence of sleep disorder in our study increased with menopause and its duration, from 19.2% in premenopause to 43.6% in perimenopause, 45.7% in early postmenopause, and 50.9% in late postmenopause. Thus, Sleep disorder is highly prevalent^37^ in menopausal women.

Sleep disorder is multi-factorial^38^. The risk factors for sleep disorder in our study such as older age, menopause, history of diabetes mellitus were in agreement with previous studies^15, 39, 40^, while we also found that baPWV and parity were independently associated with sleep disorder beyond other contributions of multiple confounders in menopausal women. We confirmed that sleep disorder was interacted with cardiovascular risks. As we all know, there has been a prevailing trend for grandparents’ upbringing in the current society in China, especially taking care for little babies in the night, thus their sleep was disturbed by crying of babies. Women with more parity may experience more stress of upbringing grandchildren. Accordingly, priority should be attached to the importance of the sleep quality in middle-aged women. And the society should call for the younger generations to encourage their mothers to embrace their own lives instead of continuing to sacrifice themselves for the next generations.

To the best of our knowledge, we may first investigate the association of sleep disorder evaluated by Pittsburgh sleep index with arterial stiffness by brachial-ankle pulse wave velocity in women with different menopausal status, and we suggest that sleep disorder contributes to the vulnerability to the arterial stiffness in peri-post menopausal not premenopausal women, independently of other classical cardiovascular risk factors. Thus, as to peri-post menopausal women, better understanding and evaluation of sleep disorder for them might provide the noteworthy candidate for easily assessing arterial stiffness, and help identify the novel risk stratification tools and therapeutic targets.

## Conclusion

In conclusion, our study demonstrated that sleep disorder was independently associated with higher arterial stiffness in menopausal women. The findings proposed that maintain good sleep quality should be attached great importance to menopausal women in order to effectively control cardiovascular risks and prevent the progression of atherosclerosis in menopausal women.

### Limitations

Several limitations deserve mention in this research. First, the inherent drawback of an observational survey may weaken the causal relationship. Secondly, systemic errors due to the baPWV tool may be produced, for its calculation of path length comes from a height-based formula for Japanese population. However, the height of Chinese is similar to that of Japanese. Finally, sleep quality ascertained by questionnaire would produce memory bias. Therefore, further longitudinal study is needed to confirm these relationships. Our team is now working on the following-up investigation.

## Method

### Study design and participants

This analytic cross-sectional study enrolled 1904 participants aged 40–60 years who visited the physical examination center in the Shanghai Sixth People’s Hospital, Shanghai Jiao Tong University School of Medicine, China, from January 2016 to November 2016. The study protocol was approved by the Ethics Committee of Shanghai Sixth People’s Hospital, and the study was performed in accordance with the approved guidelines. All the participants provided written informed consents after full explanation of the study.

The inclusion criteria were as follows: 1. Han-Chinese woman aged 40–60 years; 2. voluntary to participant in the investigation and capable of completing the questionnaire by themselves; 3. with healthy four limbs. Participants were excluded as follows: 1. with menopausal hormone treatment (MHT) and any other traditional Chinese medicine indicated for menopause within previous six months (*n* = 12); 2. history of psychiatric illness (*n* = 2); 3. history of a surgical operation in the last six months, present trauma, severe infections (*n* = 2); 4. night work shifts and irregular sleep schedule(*n* = 10); 5. with tubercle and active malignant tumor (*n* = 3); 6. current smoking (at least one pack per month for at least the previous six month) and excessive alcohol drinking within previous 6 months (at least once per week) (*n* = 8); 7. Low ankle/brachial pressure index <0.9 (*n* = 17); 8. history of myocardial infarction, angina pectoris, heart failure, arrhythmia or stroke (*n* = 14); 9. with missing lab data (*n* = 189).

### General Questionnaire

The demographic information was obtained using a general questionnaire, has been applied previously^15, 41^, which was administered by well-trained investigator through face-to-face interview including variables of age, education, marital status, parity, employment status, income per month, menopausal age, menopausal status, history of chronic disease (hypertension, diabetes mellitus).

According to the Stages of Reproductive Aging Workshop (STRAW + 10)^42^, participants were divided into premenopausal group (with regular menstrual cycle), perimenopausal group (consecutive irregularities > 7 days from their normal cycle), early postmenopausal group (absence of menstrual periods for 12 months, less than 5 years) and late postmenopausal group (absence of menstrual periods more than 5 years). Hypertension was defined as self–report of any prior diagnosis or by criteria recommended by the seventh report of the Joint National Committee on Prevention, Detection, Evaluation, and Treatment of High Blood Pressure (JNC7)^43^. Diabetes mellitus was identified by self-report, the use of hypoglycemic drugs or the following OGTT when fasting plasma glucose over 7.0 mmol/L was detected in the routine check-up^19^.

### Evaluation of Sleep quality

Participants were investigated with the validated Chinese versions Pittsburgh Sleep Quality Index (PSQI)^44^. The Pittsburgh Sleep Quality Index (PSQI) is a self-administered instrument that measures subjective sleep quality during the preceding month^44^. In brief, 18 items including in the PSQI, which are used to weigh scores based on the following 7 components: subjective sleep qualities, sleep latency, sleep duration, habitual sleep efficiency, sleep disturbance, use of sleeping medications, and daytime dysfunction. Each component ranges from 0–3 scale, which corresponds to none, <1 time/week, 1–2 times/week, ≥3 times/week, respectively. Seven components sum up to generate the total global PSQI score (range, 0–21). A PSQI score of 8 or higher is defined as sleep disorder^15^. And then patients were divided into two-score groups: PSQI score < 8 and PSQI score ≥ 8.

### Anthropometric and lab parameters

Anthropometric parameters including weight, waist and hip circumference, were recorded. Body mass index (BMI) was computed by dividing weight in kilograms by the square of their height in meters. Waist/hip ratio (WHR) was calculated by dividing the waist circumference by the hip circumference.

Blood pressure was recorded on the right arm with subjects in the supine position after a 5 minute rest using a standard sphygmomanometer; the average of 3 readings was recorded. Pulse pressure (PP) was calculated as the difference between systolic blood pressure (SBP) and diastolic blood pressure (DBP).

After an overnight fast, blood samples were tested for the analysis of serum concentration of TG, TC, HDL, LDL, and FBG.

### PWV measurement

Participants were asked to maintain in supine and rest for 5 minutes before the PWV examination, and then the cuffs were wrapped on both sides of their brachium and ankle together. The pressure waveforms were recorded simultaneously from the brachial and anterior tibial arteries by automatic waveform analyzer (BP-203RPE III, OMRON, Japan), conducted by experienced technicians who were blinded to the clinical information. We record the systolic, diastolic blood pressure and heart rate concurrently. Previous studies confirmed the validity and reproducibility of baPWV measurements^45^. The average value of baPWV is calculated of left-right sided baPWV for our analysis. We defined the upper quartile of average baPWV (≥1465.5 cm/s) as elevated arterial stiffness^19, 46^.

### Statistical analysis

All statistical analyses were performed using SPSS 22.0(IBM Corporation, Armonk, NY, USA). All the variables were tested for normal distribution by Kolmogorov-Smirnov test, Levene’s test of homogeneity of variance were further performed. They were depicted as means ± standard deviation (SD) or number (%). In addition, the continuous variables were compared using independent t-test (if data of two groups satisfy the homogeneity of variance), otherwise tested with Mann-Whitney test, whereas categorical variables were compared using χ^2^ tests or Wilcoxon rank sum test. BaPWV were stated in dichotomized form, with a threshold for 1465.5 cm/s for increased arterial stiffness (comparing the highest to the lower two tertiles). Association with pulse wave velocity and sleep disorder (PSQI ≥ 8) was computed by logistic regression analysis. We also adjusted for potential confounders. A two-sided p < 0.05 was considered to be a significant difference. Logistic regression model was assessed by the Hosmer–Lemeshow test.
